# Mechanism of the protective effect of phenylephrine pretreatment against irradiation-induced damage in the submandibular gland

**DOI:** 10.3892/etm.2012.867

**Published:** 2012-12-19

**Authors:** BIN XIANG, YA-JIE LI, XI-BO ZHAO, YANG ZOU, ZENG-GUO YU, YAN-MING ZHAO, FU-YIN ZHANG

**Affiliations:** 1 Department of Oral Medicine and Medical Research Center, Medical College, Dalian University, Dalian, Liaoning 116622;; 2 Department of Oral and Maxillofacial Surgery, First Affiliated Hospital of Dalian Medical University, Dalian, Liaoning 116011;; 3 Department of Radiotherapy, First Affiliated Hospital of Dalian Medical University, Dalian, Liaoning 116011;; 4 Department of Radiology, Second Affiliated Hospital of Harbin Medical University, Harbin, Heilongjiang 150086, P.R. China

**Keywords:** irradiation, submandibular gland, phenylephrine, proliferation, apoptosis

## Abstract

Irradiation is a fundamental treatment modality for head and neck malignancies. However, a significant drawback of irradiation treatment is the irreversible damage to salivary glands in the radiation field. Although the protective effect of phenylephrine pretreatment on salivary glands following irradiation has previously been demonstrated, the exact mechanism remains unclear. In this study, we investigated the cytoprotective mechanisms of phenylephrine pretreatment in rat submandibular glands following irradiation. Rats were locally irradiated using a linear accelerator in the head and neck region with a single dose of 20 Gy. Phenylephrine (5 mg/kg) was injected intraperitoneally 30 min prior to irradiation and the submandibular glands were collected on day 7 after irradiation. In comparison with the control group, the irradiation-only group demonstrated severe atrophy, enhanced cell proliferation and increased apoptosis. The phenylephrine-pretreated group, however, demonstrated markedly alleviated atrophy, further increased cell proliferation and decreased apoptosis compared with the irradiation-only group. The data indicated that the cytoprotective mechanisms of phenylephrine pretreatment in the submandibular gland following irradiation may be related to improved cell proliferation and inhibition of cell apoptosis.

## Introduction

Radiation delivery to the head and neck is a common treatment modality for malignancies. Salivary glands in the radiation field are severely damaged and patients experiencing reduced salivary flow suffer from considerable morbidity, including xerostomia, dental caries, mucosal infection, dysphagia and extensive discomfort (1,2). Although significant improvements, including the introduction of intensity modulated radiotherapy (IMRT), have been made for targeting radiation more precisely to the tumor and sparing normal tissues (3), the occurrence of radiation-induced sialadentitis is still inevitable (4). Therefore, it is important to increase the tolerance of normal tissues by using a radioprotector to improve patients’ quality of life.

Previously, human clinical trials and animal studies have revealed amifostine as a promising radioprotective agent that may reduce xerostomia in patients (5) and preserve salivary gland function, particularly that of the parotid glands (6,7). However, it also has clinically undesirable manifestations, including nausea, vomiting, hypotension, allergic reaction, thrombocytopenia and leucopenia (8,9). Junn *et al* also reported that varying doses of amifostine had no evident cytoprotective effects in three groups of cancer patients treated with primary chemoradiation (10). Amifostine imposes a high level of physical discomfort on patients and may lead to treatment interruption (10). Therefore, due to its high toxicity and the possibility of it protecting tumors (11), alternatives to amifostine should be explored.

It is known that the water-secretory function of the salivary gland is regulated by α-adrenoceptors and muscarinic receptors. In order to prevent xerostomia and improve the secretive function of the salivary gland following irradiation, one study pretreated rat parotid glands with cyclocytidine (an α-adrenoceptor agonist) and pilocarpine (a muscarinic receptor agonist) (12). Data revealed that cyclocytidine effectively protected the parotid gland against weight loss and flow rate reduction at early and late phases, while pilocarpine caused no significant change in any of the glandular parameters (12). Furthermore, phenylephrine, an α_1_-adrenoceptor agonist, has also shown efficacy in cytoprotection against early phase irradiation damage in the parotid gland (13). However, the exact mechanism remains unknown. The aim of this study was to investigate the molecular mechanism of cytoprotection by phenylephrine pretreatment in rat submandibular glands following irradiation.

## Materials and methods

### Animals

Male Wistar rats, weighing 230–250 g were used. They were kept in polycarbonate cages under an alternating 12 h 1ight/dark cycle. The animals were maintained on laboratory chow and water *ad libitum*. All experimental procedures were approved by the Animal Care and Use Committee and were in accordance with the Guidance of the Ministry of Public Health for the care and use of laboratory animals.

The rats were randomly divided into three groups as follows: i) the control group (n=9); ii) the irradiation-only group (n=9); and iii) the phenylephrine pretreatment group (n=9). Phenylephrine (5 mg/kg) was injected intraperitoneally 30 min prior to irradiation. The control and irradiation-only groups were administered the same volume of saline. The submandibular glands were removed on day 7 post-irradiation under standard anesthesia.

### Irradiation

Prior to irradiation, rats were anesthetized by an intraperitoneal injection of ketamine (130 mg/kg), weighed and then firmly immobilized in a box shielded with 3 mm thickness of lead, such that only the head and neck regions were exposed. The rats were locally irradiated in the region of the head and neck with a single dose of 20 Gy. We used a radiation dose that has been used in previous studies and that was expected to cause significant gland impairment (14). Irradiation was carried out with 6 MV X-rays from a Varian 23 EX linac linear accelerator (Varian Medical Systems, Palo Alto, CA, USA) at a dose rate of 5 Gy/min. The irradiation field was an area of ∼3x24 cm^2^ and the distance from the source was 100 cm. Four rats were irradiated simultaneously. Control animals were anesthetized and sham-radiated. All irradiation was carried out from 11:00 to 13:00.

### Reagents and antibodies

Phenylephrine was purchased from Sigma (St. Louis, MO, USA). Antibodies against proliferation cell nuclear antigen (PCNA) and biotin-conjugated anti-goat immunoglobulin secondary antibody were purchased from Santa Cruz Biotechnology Inc. (Santa Cruz, CA, USA). The *in situ* cell death detection kit was purchased from Roche Applied Science (Penzberg, Germany). Other chemicals and reagents were of analytical grade.

### Light microscopic observation

The submandibular gland tissues were fixed in 10% neutral-buffered formalin and processed for paraffin embedding according to a standard procedure. The submandibular gland sections were stained with hematoxylin and eosin (H&E) to evaluate the morphological changes by light microscopy.

### Immunohistochemistry

The submandibular gland tissues were fixed in 10% neutral buffered formalin and embedded in paraffin. The sections (4 *μ*m thick) were rinsed several times in phosphate-buffered saline (PBS) and then blocked with 3% hydrogen peroxide (H_2_O_2_) and 3% normal goat serum to eliminate non-specific staining. Then, the sections were incubated with goat polyclonal antibody against PCNA (1:100) overnight at 4°C. Biotin-conjugated anti-goat immunoglobulin secondary antibody was applied for 2 h at room temperature. Following incubation with streptavidin-horseradish peroxidase substrate, the slides were counterstained with hematoxylin. Negative controls were incubated with goat IgG in place of the primary antibody. The PCNA-positive cells were counted in 10 different fields in each section under ×400 magnification.

### Terminal deoxynucleotidyl transferase-mediated deoxyuridine triphosphate-biotin nick end labeling (TUNEL) staining

To detect apoptotic cells, the TUNEL method was performed using the *in situ* cell death detection kit, POD. The detection procedure was performed according to the manufacturer’s instructions. Briefly, the tissue sections were incubated with terminal deoxynucleotidyl transferase in a humidified chamber at 37°C for 1 h. A mixture of antidigoxigenin-peroxidase and substrate-chromagen was used for visualization and the sections were counterstained with hematoxylin. The nuclei of apoptotic cells were stained dark brown and counted in 10 different fields in each section under a light microscope at ×400 magnification.

### Statistical analysis

Data are expressed as mean ± standard error of the mean (SEM). Comparison of means was performed by one-way analysis of variance (ANOVA) followed by the Bonferroni test. P<0.05 was considered to indicate a statistically significant difference.

## Results

### Histopathological alterations of the submandibular gland post-irradiation

**Figure 1.**
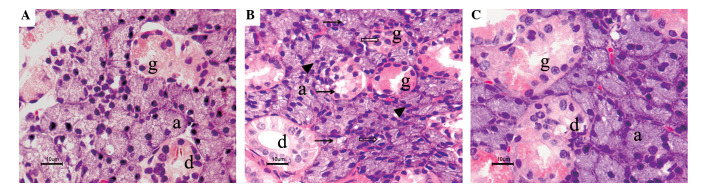
Histopathological alterations of the submandibular gland post-irradiation. All submandibular gland tissues were removed on day 7 post-irradiation. (A) Histopathological structure of the control submandibular gland. (B) The irradiated submandibular gland. (C) The phenylephrine-pretreated submandibular gland. Acinar cells are indicated by (a), ductal cells by (d) and granular convoluted tubule cells by (g). Vacuolization of acinar cells (arrow), pyknotic nuclei (arrowhead) and lysis of entire acini and granular convoluted tubules (empty arrow) were shown in the irradiated submandibular gland and mild atrophy and degeneration were shown in the phenylephrine-pretreated submandibular gland. Sections were stained with hematoxylin and eosin (H&E). Light microscopy magnification is ×400.

Normal acinar and ductal cells were observed in the control submandibular glands under a light microscope (Fig. 1A). In the irradiated submandibular glands, pathohistological changes were expressed as vacuolization of acinar cells, pyknotic nuclei and lysis of entire acini and granular convoluted tubules (Fig. 1B). However, in the phenylephrine-pretreated submandibular glands, the levels of acinar cellular atrophy and degeneration were much less than those in the irradiated glands and the morphologic manifestation was much closer to that of the control glands (Fig. 1C).

### Proliferation in the submandibular gland post-irradiation

**Figure 2.**
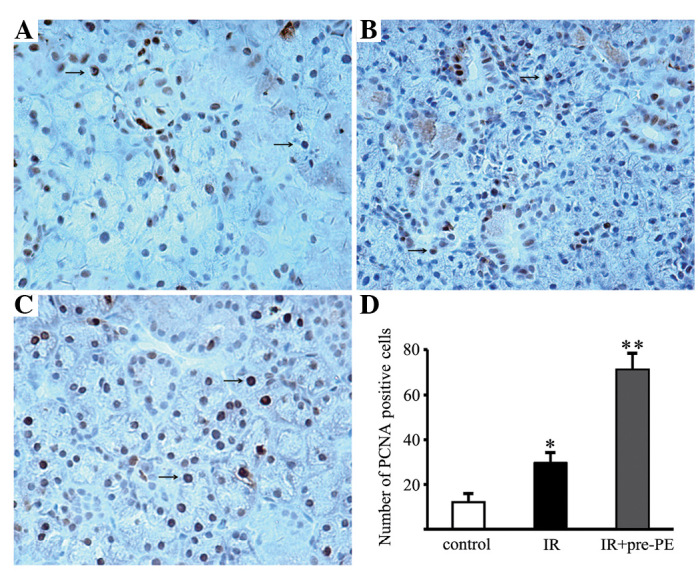
Proliferation in the submandibular gland post-irradiation. Immunohistochemical localizations of PCNA in (A) the control submandibular gland, (B) the irradiated submandibular gland and (C) the phenyl ephrine-pretreated submandibular gland are shown. PCNA-positive cells are indicated by the arrows. Light microscopy magnification is ×400. (D) Semiquantitative scoring of PCNA-positive cells. The PCNA-positive cells were counted in 10 different fields in each section under ×400 magnification. IR, the irradiated submandibular gland; IR + pre-PE, the phenylephrine-pretreated submandibular gland; PCNA, proliferation cell nuclear antigen. ^*^P<0.01, compared with the control submandibular gland. ^**^P<0.01, compared with the irradiated only submandibular gland.

A number of brown-nucleus PCNA-positive cells were identified in the ductal cells in the control submandibular gland (Fig. 2A). The numbers of PCNA-positive cells were increased in the acinar cells, granular convoluted tubules and ductal cells of the irradiated glands (Fig. 2B). They were further increased in the phenylephrine-pretreated glands (Fig. 2C). The numbers of PCNA-positive cells in the control glands, irradiated glands and phenylephrine-pretreated glands were 12.11±3.66, 29.56±4.45 and 71.22±7.17 per high-power field, respectively (Fig. 2D).

### Apoptosis in the submandibular gland post-irradiation

**Figure 3.**
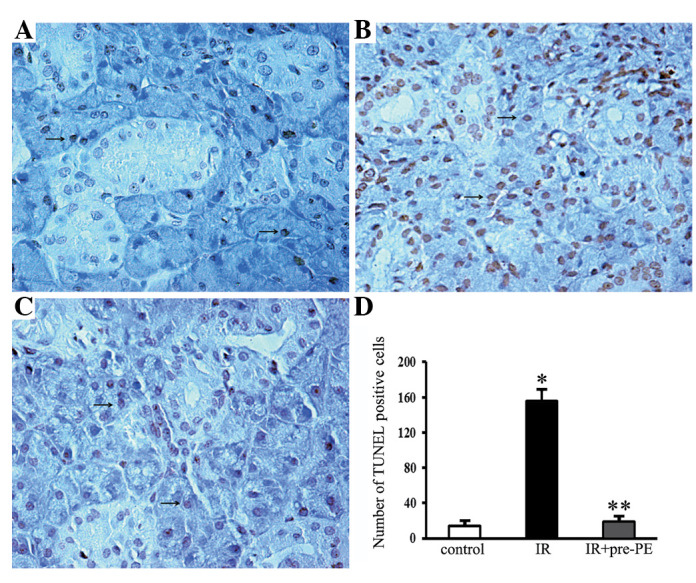
Apoptosis in the submandibular gland post-irradiation. The TUNEL-positive cells had a shrunken cell body and condensed nucleus (arrow). (A) In the control submandibular gland, TUNEL staining was rarely observed. (B) In the irradiated submandibular gland, numerous typical densely stained dark TUNEL-positive cell nuclei were observed. (C) In the phenylephrine- pretreated submandibular gland, there were markedly fewer TUNEL-positive cell nuclei than in the irradiated only gland. Light microscopy magnification is ×400. (D) Comparison of the number of TUNEL-positive cells among the different groups. IR, the irradiated submandibular gland; IR+pre-PE, the phenylephrine-pretreated submandibular gland; TUNEL, terminal deoxynucleotidyl transferase-mediated deoxyuridine triphosphate-biotin nick end labeling. ^*^P<0.01, compared with the control submandibular gland. ^**^P<0.01, compared with the irradiated only gland.

The control submandibular gland revealed very few apoptotic cells (Fig. 3A). Apoptotic activity was markedly increased in the acinar cells, intercalated cells and granular convoluted tubule cells following irradiation. TUNEL-positive cells with shrunken cell bodies and condensed nuclei were observed in the irradiated glands (Fig. 3B). Conversely, only a few TUNEL-positive cells were detected in the phenylephrine-pretreated glands (Fig. 3C). The number of TUNEL-positive cells in the control glands, irradiated glands and phenylephrine-pretreated irradiated glands were 13.67±3.39, 155.44±12.71 and 19.11±2.99 per high-power field, respectively (Fig. 3D).

## Discussion

Elucidation of the potential mechanisms underlying salivary gland radio-sensitivity has been approached by functional animal studies as well as from a molecular perspective. The most consistent observations in all animal models include significant reductions in flow rate, loss of glandular weight and loss of acinar cells (14–16). A study in rats reported a 40% reduction in salivary flow rates with single doses of 5 or 10 Gy and a 60% reduction following 15 or 20 Gy, three days after treatment (14). Additionally, studies have indicated that α-adrenoceptor agonists have the potential to be used as radio-protectants (12,13). Although the entity of the protective effect has been described and studied extensively, its underlying mechanism remains enigmatic. The results of the present study demonstrated the mechanisms underlying the cytoprotective effect of phenylephrine, which may be related to the improvement of cell proliferation and inhibition of apoptosis.

In the present study, histological staining experiments revealed that notable atrophy and degeneration occurred in the submandibular gland following irradiation, including vacuolization of acinar cells, pyknotic nuclei and lysis of entire acini and granular convoluted tubules. By contrast, the atrophy and degeneration were markedly reduced by the phenylephrine pretreatment. Our data was supported by another study which suggested that the secretory granules of acinar cells are damaged by radiation-induced lipid peroxidation, which leads to the lysis of these cells (17). Administration of α-adrenergic agonists prior to radiation has been shown to maintain partial salivary flow in rats and mice (12,17–19). Loss of serous acinar cells has been linked to reductions in salivary flow since these cells are responsible for water and protein secretion (20). Notably, there are other studies that have confirmed the protective effects of α-adrenergic agonist administration, whose results indicated that the secretory granules do not play the important role previously assumed in affecting the radio-sensitivity of the salivary glands (13). These studies suggest that the underlying mechanism of the observed improvement in salivary gland function may involve a secondary messenger-induced increase in the proliferation of salivary gland cells, resulting in the recovery of tissue following irradiation (13). The complexity of salivary gland morphology suggests the involvement of multiple pathways in the dysfunction following irradiation. Therefore, it is valuable to uncover the regulatory events and functional repair mechanisms in the cellular response of the salivary gland to irradiation.

Enhanced cell proliferation following irradiation is commonly considered as a sign of initiation of regeneration of the gland tissue (21). It is known that the activation of the α_1_-adrenoceptors by phenylephrine plays an important role in promoting cell survival and DNA repair, growth and proliferation (22,23). In order to determine the mechanism of cytoprotection, we examined the effect of phenylephrine pretreatment on cell proliferation in irradiated submandibular glands. In this study, the number of PCNA-positive cells increased in irradiated glands compared with the controls. The data was consistent with an earlier study which reported increases in proliferation in all gland compartments, reaching a maximum level at day 6 post-irradiation under 15 Gy (21). Our data also demonstrated that the number of PCNA-positive cells further increased in phenylephrine-pretreated glands. The results from our study demonstrate that the regenerative capacity of irradiated glands may be promoted by phenylephrine, resulting in improved tissue renewal following irradiation. A number of individuals have permanent salivary gland hypofunction (24), which has been attributed to the attrition of acinar cells followed by replacement with fibrotic tissue (25). Further investigation is required to determine whether phenylephrine inhibits mesenchymal fibrosis in irradiated salivary glands.

The major cause of significant acinar cell loss across animal models following irradiation has been widely debated. A study in rats quantified radiation-induced apoptosis by counting condensed nuclei and reported 2–3% apoptotic cells 6 h after treatment with a broad range of doses (2.5–25 Gy) (26). The extent of apoptosis was not dose-dependent and the authors concluded that the magnitude of apoptosis did not explain the significant loss of function (26). Conversely, radiation-induced apoptosis is dose-dependent in the parotid glands of mice, with significantly higher levels detected by immunohistochemistry against activated caspase-3 (27,28). Mouse parotid glands are ∼30% apoptotic 24 h after a single 5-Gy exposure (27,28). One study reported that 5–8% of murine submandibular gland acinar cells are apoptotic 3 days after exposure to 7.5 and 15 Gy (21), while another study observed only 2% apoptosis 24 h after irradiation with 5 Gy (28). To understand the cytoprotective mechanism of phenylephrine on the irradiated submandibular gland, we examined the level of apoptosis using TUNEL staining. An increase in the number of TUNEL-positive cells was detected in the irradiated group compared with the controls, which accords with the studies that reported apoptosis in irradiated submandibular glands (21,29,30). We also identified that the pre-administration of phenylephrine resulted in a marked decrease in the number of TUNEL-positive cells in the treated group. Previous studies have suggested that phenylephrine protects cells against apoptosis triggered by certain stressors, including injury induced by ischemia/reperfusion in the rabbit submandibular gland and hypoxia and serum deprivation in neonatal rat cardiomyocytes (31,32). In the current study, we further proved that the cytoprotective mechanism of phenylephrine in irradiated submandibular glands may be related to its anti-apoptotic efficacy.

Multiple pathways, including p53 and protein kinase C-δ (PKC-δ) regulation of apoptosis, may lead to salivary gland dysfunction following irradiation (27,28). Ionizing radiation (5 Gy) induced p53 transcriptional activation and apoptosis of mouse salivary acinar cells *in vitro* and *in vivo*(27). PKC-δ-deficient mice exhibited significantly lowered levels of radiation-induced apoptosis (1 and 5 Gy) (28). Importantly, our previous study demonstrated that phenylephrine increases the expression of phospho-PKC-ζ to improve cell survival in submandibular glands that have been damaged by ischemia/reperfusion injury (23). Since the response of the salivary glands to irradiation is complex and presumably multi-factorial, it will be important to investigate the α_1_-adrenoceptor signaling pathway to improve our understanding of irradiation-induced salivary gland dysfunction and possibly contribute to the development of new treatment strategies.

In conclusion, our findings provide the first evidence that the mechanism of the protective effect of phenylephrine is related to the improvement of cell proliferation and inhibition of apoptosis in irradiated submandibular glands. Future research into the molecular mechanism may lead to new therapeutic interventions to improve the quality of life for patients undergoing irradiation therapy for head and neck malignancies.
